# Explicit interaction information from WikiPathways in RDF facilitates drug discovery in the Open PHACTS Discovery Platform

**DOI:** 10.12688/f1000research.13197.2

**Published:** 2018-10-12

**Authors:** Ryan A. Miller, Peter Woollard, Egon L. Willighagen, Daniela Digles, Martina Kutmon, Antonis Loizou, Andra Waagmeester, Stefan Senger, Chris T. Evelo

**Affiliations:** 1 Department of Bioinformatics (BiGCaT), Maastricht University, Maastricht, The Netherlands; 2 GlaxoSmithKline (GSK), Stevenage, UK; 3 Pharmacoinformatics Research Group, Department of Pharmaceutical Chemistry, University of Vienna, Vienna, Austria; 4 Maastricht Center for Systems Biology (MaCSBio), Maastricht University, Maastricht, The Netherlands; 5 Blue Sky IT, Amsterdam, The Netherlands; 6 Micelio, Antwerp, Belgium; 7 Open PHACTS Foundation, Science Park, Cambridge, UK

**Keywords:** Open PHACTS, drug discovery, semantic, bioinformatics, WikiPathways, pathway database, API

## Abstract

Open PHACTS is a pre-competitive project to answer scientific questions developed recently by the pharmaceutical industry. Having high quality biological interaction information in the Open PHACTS Discovery Platform is needed to answer multiple pathway related questions. To address this, updated WikiPathways data has been added to the platform. This data includes information about biological interactions, such as stimulation and inhibition. The platform's Application Programming Interface (API) was extended with appropriate calls to reference these interactions.  These new methods of the Open PHACTS API are available now.

## Introduction

Targeting proteins to ideally restore normal biological processes is a common starting point in drug discovery ^1^. The Open PHACTS Discovery Platform (OPDP) was designed to help identify protein targets and information about their associations with each other ^2– 4^. The OPDP supports target identification and validation by including target-target interactions from WikiPathways ^5– 7^. Of these interaction networks, proteins sharing a downstream path allows investigation of alternative drug target combinations. Even the knowledge of which biological pathways participate in disease-related processes provides insight in the pathway topology between the targets. The importance and need of providing access to interaction information for real-world research questions was outlined in a recent Open PHACTS paper ^8^.

The Open PHACTS project was born out of the desire to integrate pharmacological data from multiple precompetitive sources to efficiently address scientific questions that cannot be answered with single data sources ^8^. It integrates data using linked data approaches ^3^ from chemical and biological sources such as ChEBI, ChEMBL, UniProt, and WikiPathways ^6^. However, the OPDP did not previously include calls to access specific up- and downstream interaction effects. This information is needed for questions related to drug repositioning and repurposing. Up- or downstream targets may be interesting alternatives with similar therapeutic effect to targets, for which it is particularly hard to develop a drug agent. Thus, finding a target that has already been drugged or is more drug tractable will be advantageous. Here we describe how to identify alternative targets in the same cellular pathway using OPDP against the WikiPathways data.

## Methods

### Implementation

The WikiPathways Resource Description Framework data (WPRDF) is released as part of the monthly releases ^5^. The native format for WikiPathways is Graphical Pathway Markup Language (GPML) based on the eXtensible Markup Language (XML) standard. The RDF export is transformed from the original GPML. In the RDF representation we use two distinct controlled vocabularies, to distinguish between the graphical notation of a pathway and the biological meanings expressed in the pathway. This is done to allow integration with other pathway repositories which use other graphical notations or none. The WikiPathways RDF also includes details about directed and undirected interactions. Directed biochemical interactions capture the source and target which are depicted as an arrow in simple pathway drawings. WikiPathways adds biological meaning to interactions with Molecular Interaction Map (MIM) interaction types, like inhibitions, enzyme catalyzed reactions, and stimulations ^9^, as well as Systems Biology Graphical Notation (SBGN) interactions ^10^. Reactome pathways in WikiPathways use SBGN interactions ^11, 12^. However, because MIM and SBGN use different drawing styles, we normalize their inhibition types into a common inhibition type, defined by the WikiPathways ontology ( https://vocabularies.wikipathways.org/wp).

The WikiPathways basic drawing tools also contain generic arrows and T-bar annotations that give the user the ability to create basic diagrams without the semantic meaning of MIM or SBGN notations. The interactions connecting these nodes are captured, but the only explicit information is that it is a directed interaction from a source to a target. To handle more complicated enzyme reaction drawings, where there is not a single line that directly connects targets in a cascade of enzymatic reactions, a query was developed that recognizes these types of reactions. However, this is not implemented in the current Open PHACTS Application Programming Interface (API).

Version 2.1 of the OPDP API contains three new calls for interactions and their pathways. The first call, */pathway/getInteractions*, returns all interactions involved in a pathway. To use this feature, the user specifies a pathway URI and OPDP returns its interactions including information about direction and the connected entities. The direction information is relayed as a starting node having a *wp:source* annotation, while the end of the interaction has the *wp:target* annotation. In its simplest form, this means that if gene product A is interacting with a gene product B, then we have *wp:source* for product A and *wp:target* for product B. However, the presented new methods also support interactions with multiple sources and targets for more complex interactions that are more accurately represented this way.

The second added call, */pathways/interactions/byEntity*, returns the direction of the interactions involving this entity. An entity is specified by a URI and can be a metabolite, protein, gene product, or RNA. API options allow the user to select only upstream or only downstream interactions. If a direction is not specified in the call, all the adjacent interactions will be retrieved regardless of their direction. The results also specify the interaction type (e.g. inhibition, stimulation, conversion). Vocabularies.wikipathways.org also identifies catalysis and binding events as well as a more generic directedInteraction in the case where the type of the interaction is not identified. This ability to select the interaction direction is specifically what allows users to answer scientific questions around upstream and downstream effects, such as those defined by Open PHACTS. The third API call is */pathways/interactions/byEntity/count* which is a helper function that returns the number of interactions for a target.

### Operation

The OPDP API calls are backed by SPARQL searches against the loaded WikiPathways RDF. The query parameters that are required or optional are given in the documentation of Open PHACTS ( https://dev.openphacts.org/docs/2.1). As in previous versions, the API uses HTTP GET to call methods and needs a (free) application ID and key (see https://dev.openphacts.org/signup) ^3^.

To ensure multiple URI schemes can be used to identify genes, proteins, and metabolites, the Open PHACTS platform uses an Identifier Mapping Service (IMS) ^6^. This ensures that people can use Ensembl, NCBI Gene, and others for genes, UniProt, Ensembl, etc. for proteins, and HMDB, ChEBI, CAS registry number, and PubChem for metabolites. Furthermore, it supports identifiers.org formatted URIs, further simplifying entering identifiers ^13^.

## Example queries

We are demonstrating the platform with three example calls. All the API calls require use of an application ID and an application key. This key and ID can be acquired by creating a free Open PHACTS account. The first example is an application to the PI3K/AKT pathway for cell growth regulation which contain important targets for cancer treatment ^14^. The AKT protein has a central role and usefully shows the API call’s ability to return connected elements with the */pathways/interactions/byEntity* and the */pathway/getInteractions* calls. The API calls can help aid drug discovery by taking a target, in this case AKT, and easily identify other connected proteins that could potentially be used as drug targets with a common downstream effect.

**Figure 1.**
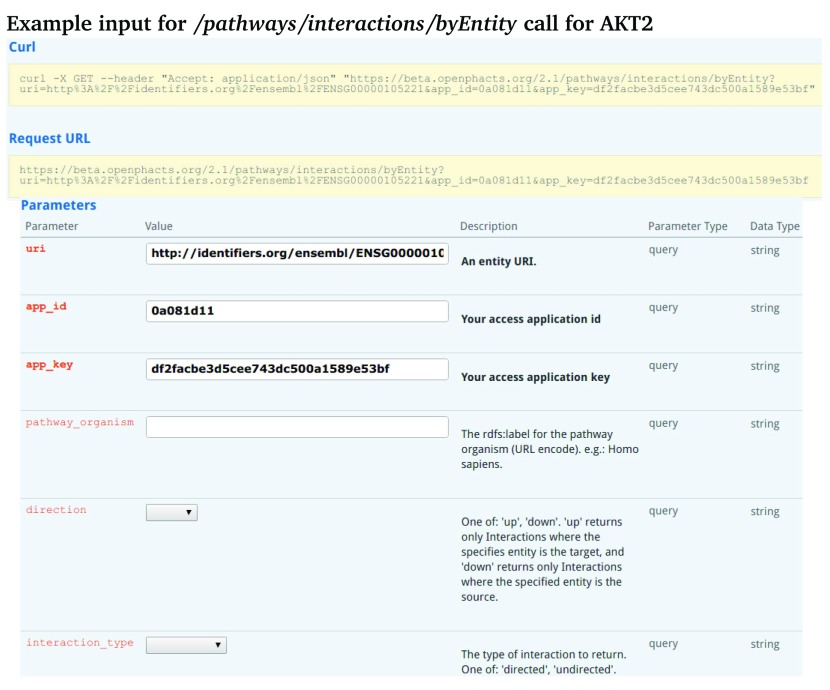
Parameters (bottom) and *curl* command (top) for the GET */pathways/interactions/byEntity* call. The GET portion tells the API to retrieve data with the associated call. It takes an entity URI, the Ensembl ID for AKT2, and returns a list interactions for AKT2. The obligatory parameters are shown in bold. Entity IDs that are acceptable for queries include Ensembl, Entrez Gene, and UniProt for genes, proteins, and RNAs. For metabolites the ID sources HMDB, ChEBI, and ChemSpider, for example, are acceptable entity IDs

**Figure 2.**
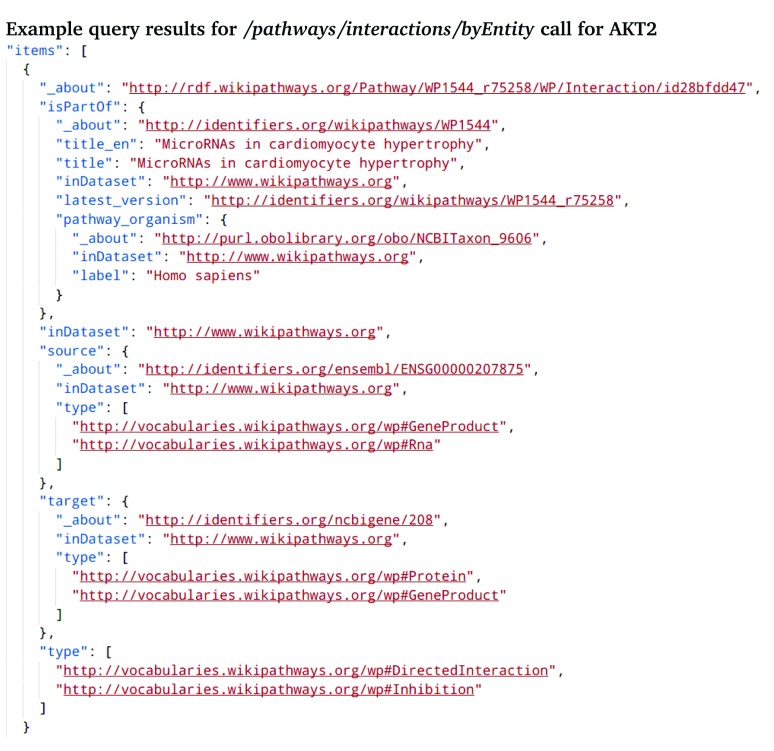
Result in the JSON format of the AKT2 query from Figure 1. The participants of the interaction are directed from source (hsa-let7b) to target (AKT2). It also shows the type of interaction (inhibition), and the biological types of the interaction participants.

**Figure 3.**
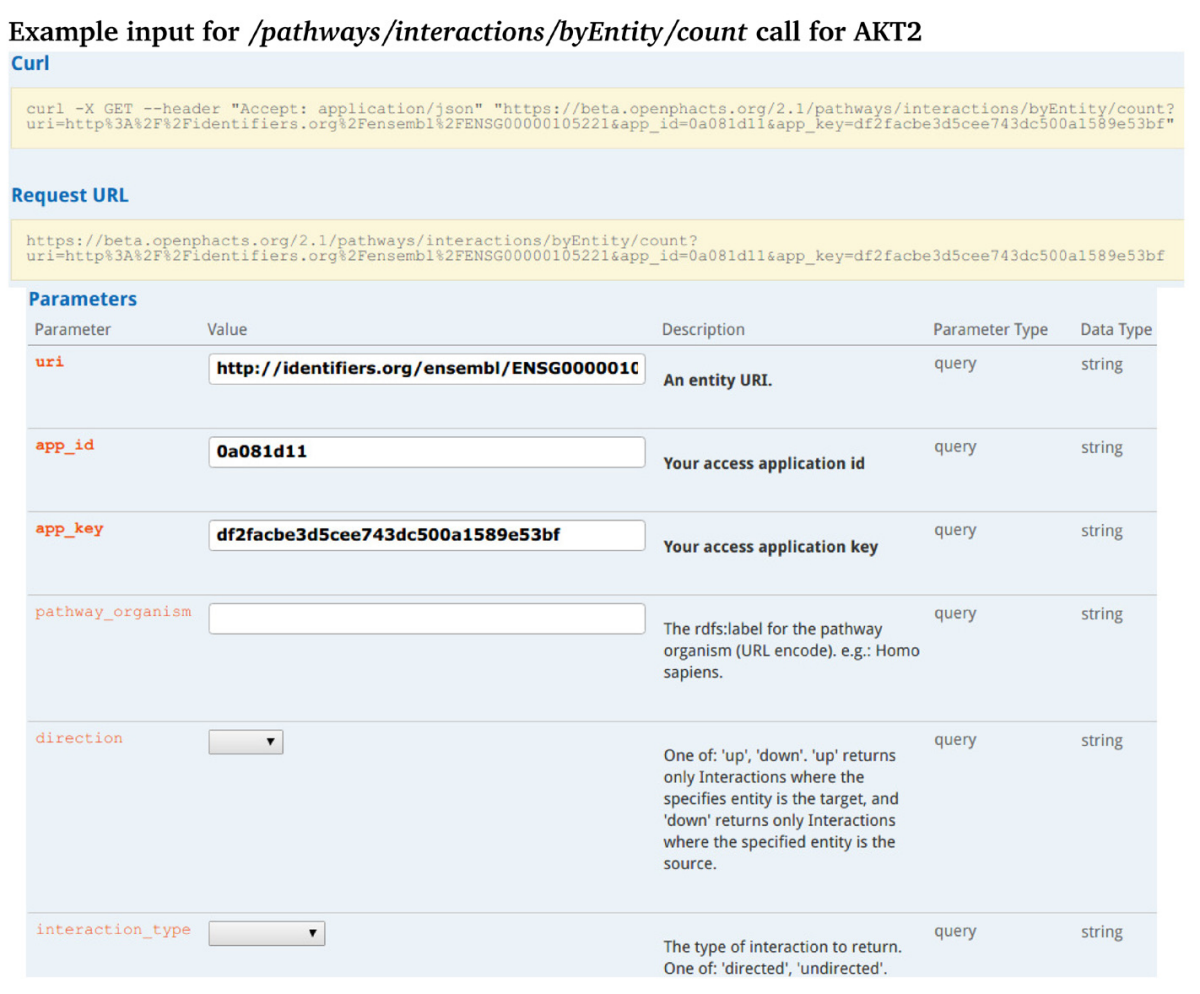
Parameters (bottom) and *curl* command (top) for the GET */pathways/interactions/byEntity/count* call. It takes a URI for an entity, in this case the Ensembl ID for AKT2 and returns a count of the interactions to which this gene product is involved. Only the entity URI, app ID, and app key are required fields. Optional parameters are pathway organism, direction, or type of interaction.

Figure 1 shows the web interface of the API call that returns the connectivity of the AKT2 target to both upstream or downstream proteins or gene products. This method allows the user to identify connections to other targets in the pathway. The results of that API call ( Figure 2) show the AKT2 interaction with microRNA. A helper method ( Figure 3): */pathways/interactions/byEntity/count* is also included. It returns the number of all interactions in which an entity is participates. This helps the user get a sense of the prevalence of the queried entity with interactions in pathways found on WikiPathways. An example result for this query can be found in Supplementary Figure 1.

**Figure 4.**
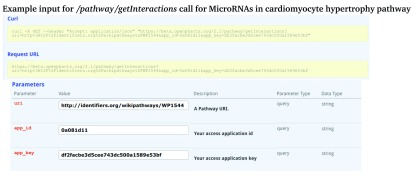
Parameters (bottom) and *curl* command (top) for the */pathway/getInteractions* call. It is intended to take the pathway URI from WikiPathways and return a list of interaction involved in that particular pathway. Pathway URI, app ID, and app key are the only required values for this call.

The other call implemented, */pathway/getInteractions* ( Figure 4), demonstrates an API call to return all interactions in the MicroRNAs in cardiomyocyte hypertrophy pathway ^15^. This pathway has interaction details for AKT, mTOR, and PI3K, which are all important targets in cancer research ^16^. For each interaction the participants are given and whether it is a directed or undirected interaction. An example result for this query can be seen in Supplementary Figure 2.

## Example workflows

In order to demonstrate the basic use of the introduced API methods, we developed two workflows, available in the Supplementary Material. One uses Python to return a file with the results in a table and the other uses a HTML webpage using the ops.js JavaScript client library ^17^. More involved workflows have been developed for KNIME and Pipeline Pilot ^18, 19^.

The Python script example uses the Open PHACTS */pathway/getInteraction* API call and prompts the user to enter a WikiPathways pathway number that they wish to query, such as 1544 for WikiPathways pathway WP1544. Invocation of the API call with the pathway identifier returns information about the directed interactions that are involved with the pathway. The information that is returned is the interaction ID used by WikiPathways, the interaction type, and URIs for the source and target of the interaction. In order to convert the URIs into something more readable, a SPARQL query is then executed to get labels, from the WikiPathways SPARQL endpoint, for the source and target of the interaction. The results are written to a file with the interaction ID, interaction type, URIs for the source and target, as well as alias IDs, the *curl* for the API call, the pathway ID used, and a number of interactions returned.

The second example uses a HTML5 webpage and the ops.js JavaScript client library to retrieve interactions for a particular gene, using the URI for the gene’s Ensembl identifier and the */pathways/interactions/byEntity* API method. The ops.js library passes the returned JSON with interaction information to a callback function, where the interacting source and target are extracted and the interacting entity determined. For each interacting entity, which may be a protein, RNA, or small compound, a call to the */pathways/interactions/byEntity/count* method is made to return the number of interaction that entity has.

## Summary

While the calls identified here are simple calls, workflow tools make it possible to take advantage of the integrative nature of the OPDP to make API calls in succession. Two such workflow tools that work with the OPDP are KNIME and Pipeline Pilot. With these tools, it is possible to perform a directional query of a target and identify alternative targets that can then be queried against the chemistry calls to identify active compounds for these alternative targets. The client libraries ops.js, ops4j, and ropenphacts also support Open PHACTS and the interaction calls for pathways. This allows users to perform API calls to the OPDP using their preferred language or platform, such as JavaScript, Java, or R.

The addition of interactions with direction information allows OPDP to answering more of the pre-defined scientific questions ^2^. The directional information allows the user to explore how proteins and gene products are connected with one another and easily access this information. This is illustrated in the example queries using the cancer target AKT.

## Software availability

Online service: https://dev.openphacts.org/docs/2.1

Latest source code is available at: https://github.com/openphacts/OPS_LinkedDataApi

Archived source code of discussed version: https://doi.org/10.5281/zenodo.1068252 ^20^

License: Apache License 2.0

## Supplementary material

Supplementary File 1. Contains two additional figures that show example API call output and contains the workflows to demonstrate the use of the APIs.
