# Supplementary material for: Explicit interaction information from WikiPathways in RDF facilitates drug discovery in the Open PHACTS Discovery Platform
Source: F1000Res. 2018 Oct 12;7:75. Originally published 2018 Jan 17. [Version 2] doi: 10.12688/f1000research.13197.2 (PMC6206606; doi:10.12688/f1000research.13197.2)
Supplement: Supplementary file 1 [file f1000research-7-17945-s0000.tgz › a5fece9c-ef75-4d29-8ae2-f3197daeb74d_Supplementary_File_1.pdf]

Supplementary Material

Example query results for */pathways/interactions/byEntity/count* call for AKT2


```
{
  "format": "linked-data-api",
  "version": "2.2",
  "result": {
    "_about": "http://beta.openphacts.org:3002/2.2/pathways/interactions/byEntity/count?
uri=http%3A%2F%2Fidentifiers.org%2Fensembl%2FENSG00000105221&app_id=0a081d11&app_key=df2facbe3d5cee743dc500a1589e53bf",
    "definition": "http://beta.openphacts.org:3002/api-config",
    "extendedMetadataVersion": "http://beta.openphacts.org:3002/2.2/pathways/interactions/byEntity/count?
uri=http%3A%2F%2Fidentifiers.org%2Fensembl%2FENSG00000105221&app_id=0a081d11&app_key=df2facbe3d5cee743dc500a1589e53bf&_metadata=all%2C
views%2Cformats%2Cexecution%2Cbindings%2Csite",
    "linkPredicate": "http://www.w3.org/2004/02/skos/core#exactMatch",
    "activeLens": "Default",
    "primaryTopic": {
      "_about": "http://www.wikipathways.org",
      "interactions_count": 2,
      "isPrimaryTopicOf": "http://beta.openphacts.org:3002/2.2/pathways/interactions/byEntity/count?
uri=http%3A%2F%2Fidentifiers.org%2Fensembl%2FENSG00000105221&app_id=0a081d11&app_key=df2facbe3d5cee743dc500a1589e53bf"
    }
  }
}
```


Supplementary Figure 1: Result in the JSON format of the AKT2 query from Figure 3. This is the count of AKT interactions with the Ensemble ID used for AKT2.

Example query results for */pathway/getInteractions* call for WikiPathways pathway 1544


```
{
    "_about": "http://rdf.wikipathways.org/Pathway/WP1544_r75258/WP/Interaction/id28bfdd47",
    "inDataset": "http://www.wikipathways.org",
  ▼ "source": {
        "_about": "http://identifiers.org/ensembl/ENSG00000207875",
        "inDataset": "http://www.wikipathways.org",
      ▼ "type": [
            "http://vocabularies.wikipathways.org/wp#GeneProduct",
            "http://vocabularies.wikipathways.org/wp#Rna"
        ]
    },
  ▼ "target": {
        "_about": "http://identifiers.org/ncbigene/208",
        "inDataset": "http://www.wikipathways.org",
      ▼ "type": [
            "http://vocabularies.wikipathways.org/wp#GeneProduct",
            "http://vocabularies.wikipathways.org/wp#Protein"
        ]
    },
    "type": "http://vocabularies.wikipathways.org/wp#DirectedInteraction"
},
```


Supplementary Figure 2: Result in the JSON format of the pathway query from Figure 4. This shows a truncated result of the *getInteractions* call for WikiPathways 1544. It also includes the same participants as was used in the specific interaction call (*/pathways/interactions/byEntity*) with the participants being AKT2 and hsa-let-7b.

Python Workflow Example

The Python workflow can be found in GitHub as `OPS_workflow.py` at the following link `https://github.com/RyAMiller/OPS_AppNote/tree/master`

```
#!/usr/bin/env python3

import sys
import urllib.request
import urllib.parse
import json
import pprint
import requests
import csv
from urllib.parse import urlparse
from SPARQLWrapper import SPARQLWrapper, XML
import xml.dom.minidom

def sparqlQ (concept):
   sparql = SPARQLWrapper("http://sparql.wikipathways.org/")
   sparql.setQuery("""
      PREFIX wp:     <http://vocabularies.wikipathways.org/wp#>
      PREFIX wprdf:  <http://rdf.wikipathways.org/>
      PREFIX dc:     <http://purl.org/dc/elements/1.1/>
      PREFIX dcterms: <http://purl.org/dc/terms/>
      PREFIX rdfs: <http://www.w3.org/2000/01/rdf-schema#>
      SELECT distinct ?label
      WHERE {
        <""" + concept + """> rdfs:label ?label
      }
   """)
   sparql.setReturnFormat(XML)
   results = sparql.query().convert()
   print(concept)

   litList = results.getElementsByTagName("literal")
   if (litList) :
      lit1 = litList[0]
      print (lit1.firstChild.wholeText)
      sparqlRes = lit1.firstChild.wholeText
      return sparqlRes
   else :
      sparqlRes = 'NA'
      return sparqlRes


OPS_api = 'df2facbe3d5cee743dc500a1589e53bf'
ops_ID = '0a081d11'
inputPWID = input('enter your pathway Id to get interactions: ')

apiID = '&app_id=' + ops_ID + '&app_key=' + OPS_api
apiFormat = '&_format=json'
f = open('getInteractionsOutput.csv', 'w')
pathwayId = 'WP' + inputPWID
queryType =
     'pathway/getInteractions?uri=http%3A%2F%2Fidentifiers.org%2Fwikipathways%2F'
     + pathwayId
url = 'https://beta.openphacts.org/2.1/' + queryType + apiID + apiFormat

print (url + '\n')
f.write(url + '\n\n')

json_obj = requests.get(url)
json_obj.raise_for_status()
data = json_obj.json()
```

```
i = 0

if isinstance(data['result']['primaryTopic']['latest_version'], (list)):
   for value in
        data['result']['primaryTopic']['latest_version'][0]['hasPart']:
      i = i + 1
      interactCount = i
      jsonvalue = json.dumps(value)
      loadedValue = json.loads(jsonvalue)
      print ('interaction\t' + value['_about'])
      f.write('interaction\t' + value['_about'] + '\n')
      print ('int type\t' + value['type'])
      f.write('int type\t' + value['type'] + '\n')

      if isinstance(loadedValue['source'], (list)):
         for values in loadedValue['source']:
            url2bencoded = values['_about']
            sparqlRes = sparqlQ(url2bencoded)
            print ('source\t' + values['_about'] + '\taliasIDs\t' + sparqlRes
                 + '\n' )
            f.write('source\t' + values['_about'] + '\taliasIDs\t' +
                 sparqlRes + '\n' )
      else:
         url2bencoded = value['source']['_about']
         sparqlRes = sparqlQ(url2bencoded)
         print ('source\t' + value['source']['_about'] + '\taliasIDs\t' +
              sparqlRes + '\n' )
         f.write('source\t' + value['source']['_about'] + '\taliasIDs\t' +
              sparqlRes + '\n' )

      if isinstance(loadedValue['target'], (list)):
         for values in loadedValue['target']:
            url2bencoded = values['_about']
            sparqlRes = sparqlQ(url2bencoded)
            print ('target\t' + values['_about'] + '\taliasIDs\t' + sparqlRes
                 + '\n\n' )
            f.write('target\t' + values['_about'] + '\taliasIDs\t' +
                 sparqlRes + '\n\n' )
      else:
         url2bencoded = value['target']['_about']
         sparqlRes = sparqlQ(url2bencoded)
         print (sparqlRes, "xxxxxxxxxxxxxxxxxxxxxxxx")
         print ('target\t' + value['target']['_about'] + '\taliasIDs\t' +
              sparqlRes + '\n\n' )
         f.write('target\t' + value['target']['_about'] + '\taliasIDs\t' +
              sparqlRes + '\n\n' )

else:
   for value in data['result']['primaryTopic']['latest_version']['hasPart']:

      i = i + 1
      interactCount = i
      jsonvalue = json.dumps(value)
      loadedValue = json.loads(jsonvalue)
      print ('interaction\t' + value['_about'])
      f.write('interaction\t' + value['_about'] + '\n')
      print ('int type\t' + value['type'])
      f.write('int type\t' + value['type'] + '\n')

      print(loadedValue)
      try:
         if isinstance(loadedValue['source'], (list)):
            for values in loadedValue['source']:
               url2bencoded = values['_about']
               sparqlRes = sparqlQ(url2bencoded)
               print ('source\t' + values['_about'] + '\taliasIDs\t' +
```

```
                sparqlRes + '\n' )
            f.write('source\t' + values['_about'] + '\taliasIDs\t' +
                sparqlRes + '\n' )
    except KeyError:
        pass

    else:
        url2bencoded = value['source']['_about']
        sparqlRes = sparqlQ(url2bencoded)
        print ('source\t' + value['source']['_about'] + '\taliasIDs\t' +
            sparqlRes + '\n' )
        f.write('source\t' + value['source']['_about'] + '\taliasIDs\t' +
            sparqlRes + '\n' )
    try:
        if isinstance(loadedValue['target'], (list)):
            for values in loadedValue['target']:
                url2bencoded = values['_about']
                sparqlRes = sparqlQ(url2bencoded)
                print ('target\t' + values['_about'] + '\taliasIDs\t' +
                    sparqlRes + '\n' )
                f.write('target\t' + values['_about'] + '\taliasIDs\t' +
                    sparqlRes + '\n\n' )
    except KeyError:
        pass

    else:
        url2bencoded = value['target']['_about']

        sparqlRes = sparqlQ(url2bencoded)
        print ('target\t' + value['target']['_about'] + '\taliasIDs\t' +
            sparqlRes + '\n\n' )
        f.write('target\t' + value['target']['_about'] + '\taliasIDs\t' +
            sparqlRes + '\n\n' )

print ('PW ID\t',inputPWID)
f.write('PW ID\t' + inputPWID + '\n')
print ('interactions for the pathway\t{}'.format(interactCount) + '\n')
f.write('interactions for the pathway\t{}'.format(interactCount))
f.close()
```

JavaScript Workflow Example

The JavaScript workflow can be found in GitHub as `interactingGenes.html` at the following link `https://github.com/RyAMiller/OPS_AppNote/tree/master`

```
<!DOCTYPE html>
<html>
<head>
  <title>Genes interacting with AKT2</title>
  <meta http-equiv="Content-Type" content="text/html; charset=UTF-8">

  <!-- ops.js, d3.js, and dependencies -->
  <script type="text/javascript"
       src="https://egonw.github.io/pils/lib/purl.js"></script>
  <script type="text/javascript"
       src="https://egonw.github.io/pils/lib/jquery-1.9.1.min.js"></script>
  <script type="text/javascript"
       src="https://egonw.github.io/pils/src/combined.js"></script>

</head>

<body>
  <h3>Gene</h3>
  <div id="uri"></div>
```

```
<p>
<table>
  <thead>
    <tr>
      <td><b>Interaction Gene</b></td>
      <td><b>Database</b></td>
      <td><b>Number of Interaction</b></td>
    </tr>
  </thead>
  <tbody id="list">
  </tbody>
</table>
</p>

<!-- dynamically create a table with type information -->
<script type="text/javascript">

var query = location.search.substr(1);
var result = {};
query.split("&").forEach(function(part) {
  var item = part.split("=");
  result[item[0]] = decodeURIComponent(item[1]);
});

var queryGene = 'http://identifiers.org/ensembl/ENSG00000105221'
if (result["uri"]) queryGene = result["uri"]
document.getElementById("uri").innerText = "Query: " + queryGene
var pathwayService = new PathwaySearch(
  "https://beta.openphacts.org/2.1", "yourAppID", "yourAppKey"
);
// results return asynchronously from the
     pathwayService.getInteractionsByEntity()
// call and are handled by the method
var handleInteractingGenes = function(success, status, interactionData) {
  if (success && status == 200) {
    var itemCount = interactionData.items.length;
    var uniqueGenes = []
    for (var i = 0; i < itemCount; i++) {
      var interaction = interactionData.items[i]
      var rowNode = document.createElement("tr");
      if (interaction.source._about != queryGene) otherGeneURI =
           interaction.source._about
      if (interaction.target._about != queryGene) otherGeneURI =
           interaction.target._about
      var database = otherGeneURI.split("/")[3]
      var otherDatabase = document.createElement("td");
      var otherGene = otherGeneURI.split("/")[4]
      var otherNode = document.createElement("td");
      if (!(uniqueGenes.includes(otherGene))) {
        otherNode.innerText = otherGene
        otherDatabase.innerText = database
        rowNode.setAttribute("id", otherGene)
        rowNode.appendChild(otherNode)
        rowNode.appendChild(otherDatabase)
        document.getElementById("list").appendChild(rowNode);
        pathwayService.countInteractionsByEntity(
          otherGeneURI, null, null, null, handleInteractionCount
        );
        uniqueGenes.push(otherGene)
      }
    }
  }
};
// results return asynchronously from the
     pathwayService.countInteractionsByEntity()
// call and are handled by the method
```

```
  var handleInteractionCount = function(success, status, interactionData) {
    if (success && status == 200) {
      var geneURI =
           interactionData.primaryTopic.isPrimaryTopicOf.split("uri=")[1]
      geneURI = geneURI.replace(new RegExp("%2F", 'g'), "/")
      geneURI = geneURI.replace("%3A", ":")
      var geneid = geneURI.split("/")[4]
      var count = interactionData.primaryTopic.interactions_count
      var otherNode = document.createElement("td");
      var otherLink = document.createElement("a");
      otherLink.setAttribute("href", "?uri=" + geneURI)
      otherLink.innerText = count
      otherNode.appendChild(otherLink)
      document.getElementById(geneid).appendChild(otherNode);
    }
  };
  pathwayService.getInteractionsByEntity(
    queryGene, null, null, null, 0, 1000, null,
    handleInteractingGenes
  );
  </script>
</body>
</html>
```
