# Supplementary material for: Explicit interaction information from WikiPathways in RDF facilitates drug discovery in the Open PHACTS Discovery Platform
Source: F1000Res. 2018 Oct 12;7:75. Originally published 2018 Jan 17. [Version 2] doi: 10.12688/f1000research.13197.2 (PMC6206606; doi:10.12688/f1000research.13197.2)
Supplement: Peer review history

# Peer review: Referee response for version 2

**DOI:** 10.5256/f1000research.17945.r39411

**Published:** 2018-10-23

**Authors:** Augustin Luna

**Affiliations:** 1 Dana-Farber Cancer Institute, Boston, MA, USA

The authors satisfactorily addressed my concerns.

I have read this submission. I believe that I have an appropriate level of expertise to confirm that it is of an acceptable scientific standard.

# Peer review: Referee response for version 2

**DOI:** 10.5256/f1000research.17945.r39412

**Published:** 2018-10-19

**Authors:** Yi-An Chen

**Affiliations:** 1 National Institutes of Biomedical Innovation, Health and Nutrition, Osaka, Japan

The author has addressed all the issues. I have no further comments.

I have read this submission. I believe that I have an appropriate level of expertise to confirm that it is of an acceptable scientific standard.

# Peer review: Referee response for version 1

**DOI:** 10.5256/f1000research.14318.r32325

**Published:** 2018-04-03

**Authors:** Yi-An Chen

**Affiliations:** 1 National Institutes of Biomedical Innovation, Health and Nutrition, Osaka, Japan

The authors add new features to the Open PHACTS APIs which facilitate querying reactions in WikiPathways.

 Some comments:

1. In the first example, the query for AKT2 in the figure 1 uses http://identifiers.org/ensembl/ENSG00000105221 but the result in the figure 2 shows http://identifiers.org/ncbigene/208. I guess the API should be able to accept different commonly used identifiers, however, either the article or the web interface mention about the acceptable identifiers. In addition, It would be nice if the input of the query accepts simple identifiers, instead of constructing the full URI.
2. The result in figure 2 is difficult to interpret. It would be nice to expand the query to get the basic information for those interacting entities. For example, the gene symbol or the compound name.
3. The examples in figure 3 and figure 4 didn't show the query results.
4. Simple queries in the examples may be difficult to show the usefulness of the new implemented APIs. A more sophisticated application which contains either bulk query or pipeline query should be helpful for the readers to understand the demand of using the APIs.

I have read this submission. I believe that I have an appropriate level of expertise to confirm that it is of an acceptable scientific standard, however I have significant reservations, as outlined above.

# Response

**Published:** 2018-10-11

**Authors:** Ryan Miller

**Affiliations:** Maastricht University, The Netherlands

**1.  The Open PHACTS API includes the Identifier Mapping Service component which allows use of many identifier schemes, as long as suitable link sets are available (at http://data.openphacts.org/1.5/ims/linksets/). These sets include many of the popular gene, protein, and metabolite identifier:for genes, proteins, and RNAs Ensembl, Entrez Gene, UniProt, and others; for metabolites the ID sources can include HMDB, ChEBI, PubChem, CAS registry numbers, and ChemSpider.**

****

**2.  Retrieving specific gene symbol or compound names aside from the identifier URI, resource, and identifier can be part of a workflow in workflow tools like KNIME or Pipeline Pilot.  We did create a simple workflow to address this. In this example, we use the /pathway/getInteractions API call to return the directed interactions for a pathway and the resulting IDs for the interaction and then returned the more human readable labels for the URIs.**

****

**3.  Additional example output figures were added to the supplementary materials section to reflect the addition of example query results for the remaining calls.**

****

**4.  We added an Example Workflows section describing two simple workflows. More complex applications and workflows have been recently published for KNIME and Pipeline Pilot, which we added as reference 19.**

# Peer review: Referee response for version 1

**DOI:** 10.5256/f1000research.14318.r29933

**Published:** 2018-01-24

**Authors:** Augustin Luna

**Affiliations:** 1 Dana-Farber Cancer Institute, Boston, MA, USA

The tool provides additional features to the Open PHACTS platform to simplify the access of interaction information from WikiPathways. Some comments: 

- The authors go into some detail about the MIM/SBGN representations used by WikiPathways. To my understanding, WikiPathways makes use of the format GPML as a data storage format. Some description of the relationship of GPML and the WPRDF format would be nice for readers to understand the data processing involved. 
- Looking at the code repository, the API interface seems to be built using Swagger. Is there the plan to provide supported client packages to access the API from various programming languages? Are users encouraged to produce their own using the swagger-codegen ( https://github.com/swagger-api/swagger-codegen) project? Have the authors had any experience with these auto-generated packages in the context of their API? 
- The sentence "However, the new methods also support interactions with multiple sources and targets.", are these the methods that are elsewhere described as not implemented currently?
- What entity IDs are acceptable? The examples show ENSEMBL is this the only acceptable ID source? Does the documentation provide any examples for ID conversion from much more common gene symbols, for example?
- I assume that when "up" or "down" interactions are not specified for return then both are returned is this correct? 
- Are the example interactions (i.e. inhibition, stimulation, conversion) the only ones available in the API? If not, is there a listing of the interaction types with description of the interaction type. 
- It would be more clear to just state the call function name rather than "the first and third calls" and "the api call" in the example queries section. 
- Additional examples of the result output for all described function calls would be helpful for the described API calls. 
- Additional examples that highlight the integrative nature of Open PHACTS would be nice. For example, to show how results of the currently described API calls can paired the other data available, such as the CHEBI data from the platform.

I have read this submission. I believe that I have an appropriate level of expertise to confirm that it is of an acceptable scientific standard, however I have significant reservations, as outlined above.

# Response

**Published:** 2018-10-11

**Authors:** Ryan Miller

**Affiliations:** Maastricht University, The Netherlands

1. **WikiPathways is represented using GPML (Graphical Pathway Markup Language), which is a modified XML format.  The WikiPathways RDF is divided into two divisions, the GPML and the WPRDF. The GPML side is the portion that is responsible for the drawing and graphical elements.  The WPRDF side is there to describe the semantic elements of the RDF.  We have updated the Methods section and clarified this in the first “Implementation” paragraph.**
2. **The API is REST-like and dedicated clients are not required. However, a few client libraries are already available for various languages and platforms: JavaScript (ops.js), Java (ops4j), ropenphacts, and KNIME nodes. Personal experience with auto-generated packages has been negative, but based on code generation in general, not the Open PHACTS API.  Supported libraries are referenced in the summary sections along with the workflow environments.**
3. **We intended to clarify that this simple example referred to single-source, single-target interactions, but that the framework also supports interactions with more than one source and/or target. Such complex interactions occur frequently.** **The last sentence of the third paragraph under Methods: Implementation has been updated to make this more clear.**
4. **The Open PHACTS API includes the Identifier Mapping Service component which allows use of many identifier schemes, as long as suitable link sets are available (at http://data.openphacts.org/1.5/ims/linksets/). These sets include many of the popular gene, protein, and metabolite identifier:for genes, proteins, and RNAs Ensembl, Entrez Gene, UniProt, and others; for metabolites the ID sources can include HMDB, ChEBI, PubChem, CAS registry numbers, and ChemSpider. The caption of Figure 1 has been extended and a paragraph added to Operation section to reflect this.**
5. **Yes, you are correct: if the user does not specify the directions for the interactions, all the immediate interactions are retrieved regardless of their direction. This can be found in the last paragraph of the Methods section and updated here to clarify.**
6. **Vocabularies.wikipathways.org also identifies catalysis and binding events as well as a more generic directedInteraction in the case where the type of the interaction is not identified. This can be found in the last paragraph of the Methods: Implementation.**
7. **The text in the under Example Queries section has been updated to use the names of the API calls used.**
8. **Additional figures that show example outputs for the remaining figures can be found in the supplemental info section.**
9. **Workflow tools make it possible to take advantage of the integrative nature of the OPDP to make API calls in succession.  With these tools, it is possible to perform a directional query of a target and identify alternative targets that can then be queried against the chemistry calls to identify active compounds for these alternative targets.  An Example Workflows section has been added describing two simple workflows and the code for the workflows can be found in the supplementary information.**
